# Aligning Alternative Proteins with Consumer Values in Germany: A Values-Centric Communication Framework

**DOI:** 10.3390/foods14244322

**Published:** 2025-12-15

**Authors:** Alya Alismaili, Lena Böhler, Sonja Floto-Stammen

**Affiliations:** Research Group Business Innovation, Fontys University of Applied Sciences, 5912 BG Venlo, The Netherlands

**Keywords:** values-centric communication, Schwartz’s value theory, alternative proteins, packaging communication

## Abstract

The transition to sustainable food systems requires communication strategies that resonate with consumers’ values, not only technological innovation. This study examines how values-centric communication can shape German consumers’ responses to alternative proteins, focusing on insect-based snacks. A desk-based synthesis of recent studies, guided by Schwartz’s value theory, identified Tradition and Security as dominant drivers of food choice and yielded five communication requirements: Cultural familiarity, Emotional safety, Simplicity and clarity, Trust and credibility, and Routine integration. These were operationalised into communication guidelines and short on-pack claims, which were applied to a refined packaging prototype. An exploratory focus group (N = 7) then compared reactions to the original versus the refined packaging, analysed using McGuire’s communication–persuasion stages. Within this small exploratory group, participants reported that familiar formats, a reassuring tone, clear visual hierarchy, and salient trust cues made them more willing to consider trying the product, whereas information overload, claim–image incongruence, value-incongruent brand naming, and delayed recognition of insect content appeared to impede acceptance. The study contributes an integrative analytic lens combining Schwartz’s value theory with McGuire’s model and a set of testable guidelines for value-aligned food communication. Because the empirical evidence is based on a single small student focus group with fixed presentation order, bundled manipulations, and hypothetical intentions, these results are exploratory and self-reported and should be interpreted cautiously; future research should employ counterbalanced factorial designs with behavioural outcomes.

## 1. Introduction

Food systems are major contributors to environmental degradation and public-health burdens [1,2]. Recent estimates attribute roughly one-third of global greenhouse gas emissions to the food system, alongside substantial land-use change, biodiversity loss, and water stress [2,3,4,5]. In response, alternative proteins—including plant-based, fermented, cultivated, and insect-based options—are advanced as lower-impact substitutes for conventional meat [6,7,8,9,10]. Yet, despite rapid supply-side innovation and growing availability, consumer adoption in Western markets such as Germany remains limited [7,8,11,12]. Prior work suggests that hesitancy often reflects how such products are positioned and communicated, rather than their intrinsic properties [13,14].

A sizeable literature base shows that disgust, unfamiliarity and scepticism are salient affective barriers, especially for insect-based and other novel proteins [8,15,16,17,18]. Messaging that relies predominantly on rational appeals (e.g., environmental impact, nutrition) often fails to engage the emotional and cultural dimensions of everyday food choice [14,19,20]. Cross-country comparisons further indicate that both baseline disgust and the effects of explicit ingredient disclosure vary across markets, underscoring the need for country-specific analyses of consumer responses. In the German context, this may reflect a misalignment between innovation-centred frames and consumers’ value orientations.

Guided by Schwartz’s Theory of Basic Human Values [21], we focus on Tradition and Security, which prior research identifies as salient in German food behaviour, emphasising familiarity, routine, predictability, and reassurance [22,23]. To assess how values-centric messages may operate across the persuasion process, we adopt McGuire’s communication–persuasion model (Attention, Comprehension, Acceptance, Retention, Action) as an analytic scaffold [24,25]. This sequential structure is particularly suited to food packaging, where front-of-pack cues must first capture attention and basic understanding before they can influence acceptance, retention, and eventual action. Integrating these frameworks enables a theoretically grounded account of what messages must achieve (value activation) and when in the communication sequence they matter.

In line with Schwartz’s Theory of Basic Human Values [21], we understand *consumer values* as broad, trans-situational goals that serve as guiding principles in people’s lives and shape attitudes and behaviour across contexts. In this paper, we focus on Tradition and Security, which emphasise familiarity, routine, predictability, and reassurance, and have been shown to be particularly salient in German food behaviour [22,23]. We situate this work within the broader discussion on sustainable food systems, understood as food systems that provide food and nutrition for all while respecting planetary boundaries and supporting social and economic well-being over time [1,2,3,4,5]. Within this context, we use insect-based snacks as a prototypical case of novel protein products: ready-to-eat packaged snacks in which insects or insect-derived ingredients constitute a primary protein source and which are known to elicit strong affective and cultural reactions in Western markets [8,15,16,17,18,26].

This study makes three contributions. First, based on a desk-based synthesis of recent literature using a targeted, structured search with strict inclusion criteria (German samples, explicit value measures, 2021–2025; see Section 2.1), we articulate five communication requirements that translate Tradition and Security into designable goals: Cultural familiarity, Emotional safety, Simplicity and clarity, Trust and credibility, and Routine integration. Second, we operationalise these requirements into actionable values-centric communication guidelines and short on-pack claims, bridging theory and execution. Third, we provide exploratory evidence from a focus group with German students comparing reactions to an original versus a refined, values-centric packaging prototype for an insect-based snack—a stringent test case given cultural unfamiliarity and high aversion potential [16,26].

The study is guided by the following research question: How can values-centric communication guidelines for companies offering protein alternatives in Germany better align messaging with consumer values? We implement a sequential design: (i) identify dominant values and derive communication requirements via desk synthesis; (ii) translate requirements into guidelines and claims; and (iii) examine consumer responses using McGuire’s stages in an exploratory focus group. Our focus on Germany responds to evidence that Tradition and Security frequently shape situational and identity-related food decisions [22,23], suggesting that values-centric communication may be pivotal for acceptance.

By combining values theory with a stage-based persuasion framework, the paper offers a coherent, testable approach to designing values-centric communication for alternative proteins. The proposed requirements and guidelines are intended as hypotheses for validation in larger, controlled studies, while also providing immediate structure for practitioners seeking to align packaging and messaging with culturally embedded consumer values in the German market.

## 2. Materials and Methods

This study employed an exploratory qualitative design to examine how values-centric communication can shape consumer perceptions of alternative proteins in Germany. A qualitative approach was selected because values, emotions, and cultural associations related to food are complex, subjective phenomena that cannot be adequately captured through quantitative measures alone. The overarching research question guiding this study was:

*How can values-centric communication guidelines for companies offering protein alternatives in Germany better align messaging with consumer values?*

A sequential design was implemented in four phases. First, consumer values relevant to eating behaviour in Germany were identified through a structured desk review. Second, these values were translated into communication guidelines. Third, guidelines were operationalised into concrete packaging claims. Finally, consumer responses to these claims were explored in a focus group.

### 2.1. Secondary Data Analysis

#### 2.1.1. Identification of Consumer Values

To identify consumer values relevant to eating behaviour in Germany and to enhance transparency, we conducted a structured desk review following the core stages of the PRISMA framework (identification, screening, eligibility, inclusion). This was conceived as a targeted, structured review rather than a full systematic review, with the aim of synthesising recent, Germany-relevant evidence on values and alternative proteins.

Searches were carried out in PubMed, ScienceDirect, and Google Scholar from 2021 to 2025. We combined terms related to consumer values, eating behaviour, geography, and novel protein sources using Boolean operators, for example: (“consumer values” OR “food values”) AND (“eating behaviour” OR “food choice”) AND (“Germany” OR “Europe”) AND (“alternative proteins” OR “novel foods”). All records were exported into Excel (Microsoft Corporation, Redmond, WA, USA; Microsoft Excel 365), where duplicates and clearly irrelevant publication types (e.g., editorials, purely technological papers without consumer data) were removed prior to screening.

In the screening stage, titles and abstracts were checked for relevance to food-related consumer behaviour, emotional or value-based drivers, and German or German-specific European samples. This process yielded 73 full texts for eligibility assessment out of an initial 185 records identified across databases. Full texts were then evaluated against predefined inclusion and exclusion criteria; any uncertainties were resolved through discussion within the research team. Studies were included if they (i) reported empirical data (quantitative, qualitative, or mixed methods) on food-related consumer behaviour, (ii) were published in English between 2021 and 2025, (iii) included German samples or reported Germany-specific results within broader European studies, and (iv) explicitly operationalised personal values or closely related constructs (e.g., Tradition, Security, familiarity, reassurance). We excluded work published before 2021, papers focusing solely on technological or production processes without consumer data, studies without human participants, and texts with insufficient methodological transparency or that discussed attitudes or beliefs without a values construct. Applying these criteria, four studies were retained for the synthesis. The relatively narrow 2021–2025 window and strict criteria resulted in a small evidence base and may bias the synthesis towards more recent publications; this trade-off was accepted to ensure recency and manageability.

The retained studies were coded using Schwartz’s Theory of Basic Human Values, which provided the conceptual framework for analysis. A thematic synthesis identified recurring value drivers and mapped them onto Schwartz’s categories, with particular emphasis on Tradition (e.g., family meals, home cooking, inherited practices) and Security (e.g., safe, reliable everyday foods, reassurance). These four studies formed the empirical value base for answering the research question and for deriving the values-centric communication guidelines developed in the subsequent phases.

To triangulate and assess transferability to our context, we additionally drew on unpublished, practice-based evidence from exploratory focus groups conducted by the Fontys Research Group between 2023 and 2025 (N ≈ 200 participants across students, educators, and company representatives). These internal data were analysed qualitatively and used as supporting, non-review evidence; they did not enter the formal PRISMA study count. The focus groups converged with the review in highlighting Tradition and Security as dominant influences in food choices, thereby reinforcing the value labels and operationalisations used in the study.

#### 2.1.2. Development of Communication Guidelines

Insights from this review were complemented by Schmeltz’s conceptual work on value-based communication [27]. While not a formal methodology, Schmeltz provides a theoretical foundation for understanding how consumer values can be reflected in messaging. Based on this perspective and the thematic synthesis of the reviewed literature, five provisional communication requirements were derived: cultural familiarity, emotional safety, simplicity and clarity, trust and credibility, and routine integration. The initial coding of value-related themes in the included studies was conducted by the first author and then reviewed and refined in discussion with the third author until consensus on the five overarching requirements was reached. These categories served as the conceptual basis for the communication guidelines.

#### 2.1.3. Formulation of Packaging Claims

The guidelines were then operationalised into concrete packaging claims by synthesising findings from peer-reviewed studies on consumer behaviour, packaging communication, and responses to food innovations published between 2020 and 2025. Five operational rules were established to ensure consistency: culturally familiar language, emotionally reassuring tone, emphasis on simplicity, inclusion of trust-building cues, and alignment with consumer identity.

Insect-based protein was chosen as the test case due to its cultural unfamiliarity and high resistance in Western contexts [16]. Claims were applied to the neutral baseline packaging of *Catch Your Bug* (Marco Schebesta, Neu-Ulm, Germany), a minimally designed snack brand.

**Figure 1:**
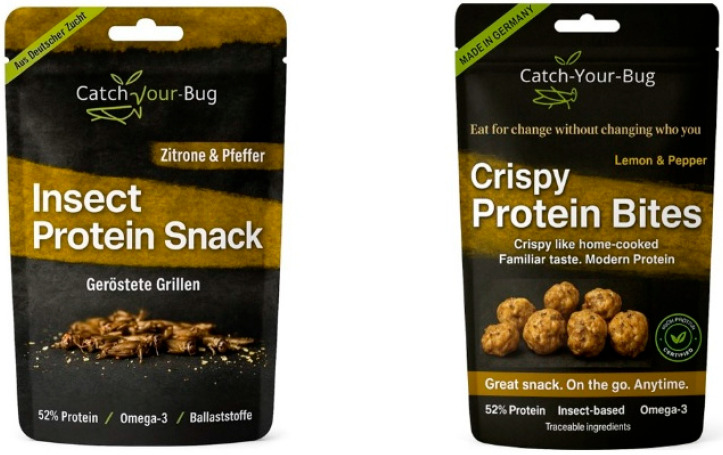
Values-centric guidelines applied to the refined version (**right**) and compared to the original (**left**) packaging. Reproduced with permission from Catch-your-Bug.

Packaging stimuli were generated using Generative AI tools. For Stimulus A (original packaging), ChatGPT Plus (OpenAI, San Francisco, CA, USA; https://openai.com/index/chatgpt-plus/, accessed on 1 March 2025) was used to reproduce and refine the visual layout and clarity of the original Catch Your Bug packaging without adding values-based elements. A high-resolution photograph of the original pack served as the visual reference for this digital replica. Prompts specified a minimalistic layout, clear hierarchy between brand name, flavour description, and protein content, and the preservation of the brand’s existing visual identity. For Stimulus B (enhanced packaging), DALL·E (OpenAI, San Francisco, CA, USA; https://openai.com/index/dall-e-3/, accessed on 1 March 2025) was used to generate a neutral, appetising product image and basic front-of-pack layout, based on short textual descriptions of the desired format (bite-sized snack pieces), style (non-cartoon, clean), and colour palette. Values-based claims and icons reflecting the five communication guidelines (Cultural familiarity, Emotional safety, Simplicity and clarity, Trust and credibility, Routine integration) were then added and positioned in a stepwise, prompt-based process (e.g., replacing the insect illustration with bite-sized snack pieces and updating headlines and subclaims) using ChatGPT Plus to ensure visual consistency between Stimuli A and B. All AI-generated outputs were reviewed and edited by the authors to ensure accuracy, clarity, and alignment with the study objectives (see Figure 1). All AI outputs were generated within licensed tools and used solely as research mock-ups; the *Catch Your Bug* brand assets were included with the explicit consent of the brand owner and are not used for any commercial purpose in this study.

### 2.2. Exploratory Focus Group

Primary data were collected through a single exploratory focus group with seven participants. Focus groups are well suited to exploring emotional and cognitive responses to novel stimuli [28]. Purposive sampling was used to recruit German residents aged 19–21 who consumed animal-based products but had no prior experience with insect-based foods. This age group was targeted because young consumers are often more open to food innovations [29]. Fluency in English was required to eliminate potential translation effects in wording, tone, and emotional nuance of packaging communication, which were discussed in English during the session. This criterion ensured consistent interpretation of claims but may introduce selection bias toward internationally oriented students. As a result, the group should be regarded as a convenience, exploratory probe of young, English-speaking German students rather than representative evidence for German consumers more broadly.

Participants were recruited via a university mailing list and screened through an online questionnaire to confirm eligibility. The final sample comprised four female and three male undergraduate students. Each participant received a €15 voucher as compensation. A single focus group was deemed appropriate given the exploratory aim of the study, which prioritised depth of insight over representativeness.

#### 2.2.1. Stimulus Preparation, Procedure, and Analysis

Two packaging versions were presented: a neutral baseline and a refined version featuring values-based claims and culturally familiar imagery (see Figure 1). The original Cath Your Bug packaging served as the baseline design but both were digitally designed to minimise confounding variables, ensuring that verbal communication constituted the primary manipulated element.

The 60-min session took place in a neutral classroom setting and followed a semi-structured topic guide based on McGuire’s Communication–Persuasion Matrix (Attention, Comprehension, Acceptance, Retention, Action), which structured questions about both packaging versions. Participants first viewed the original packaging, followed by the refined version after a short break.

Discussions were audio-recorded, transcribed verbatim, and analysed using qualitative content analysis in a framework-based approach. Coding followed McGuire’s stages deductively as main categories (Attention, Comprehension, Acceptance, Retention, Action) and was further categorised by emotional tone. Coding was conducted iteratively, with repeated comparison of emerging categories against the raw text and refinement of codes across several cycles. The initial coding was conducted in Excel (Microsoft Corporation, Redmond, WA, USA; Microsoft Excel 365) by the lead researcher with text segments organised in a coding matrix and assigned to stage and tone codes. To enhance reliability, a 20% subset of the transcript was independently coded by a second researcher, yielding an inter-coder agreement of 87%. Discrepancies in code assignment were discussed until full consensus was reached and, where necessary, code definitions were refined before being applied to the full dataset. Discrepancies were resolved through discussion, and an audit trail of coding decisions was maintained to ensure transparency. The lead researcher’s background in consumer behaviour and alternative proteins may have sensitised them to particular themes; this potential influence was addressed through explicit use of McGuire’s coding frame, double-coding of a subset, and peer debriefing, but cannot be fully eliminated.

#### 2.2.2. Ethical Considerations and Limitations

Written informed consent was obtained from all participants prior to data collection. Confidentiality was assured, and participants were anonymised in transcripts.

This study is exploratory and subject to several constraints. Reliance on secondary data in earlier phases limits originality and may import biases from prior research. The focus group was small and demographically narrow, providing rich but non-generalisable insights. Stimulus materials created with AI may contain stylistic artefacts beyond the intended manipulations. Despite inter-coder checks, coding was conducted primarily by one researcher, which may have introduced subjectivity. Nevertheless, the sequential design offers a coherent framework for linking consumer values to communication strategies and provides preliminary insights into how value-based messaging may influence perceptions of alternative proteins.

## 3. Results

This study followed a sequential design, progressing from a synthesis of existing evidence to exploratory testing of values-centric claims in a focus group. The results reported here concern the desk-based synthesis, the derivation of communication requirements and guidelines, their application to packaging claims, and the exploratory consumer responses.

### 3.1. Core Consumer Values

A thematic synthesis of recent studies (Table 1) identified Tradition and Security as the two most consistent values shaping food-related behaviour in Germany. The four studies comprise one nationally representative survey of German adults, one qualitative study with young adults, one quantitative study on food choices during inflation in a broad demographic sample, and one study focusing on health-conscious individuals (see Table 1 for an overview).

While Sustainability and Health were frequently discussed, they tended to be secondary to the cultural and emotional anchors of Tradition and Security.

In Schwartz’s framework, Tradition denotes respect for inherited practices and social norms. In the German context, this value is apparent in eating routines established through family and cultural habitus. Nationally representative evidence indicates the continued centrality of meat to the notion of a “proper meal,” with a high share of regular meat consumption [23]. Qualitative work similarly emphasises continuity—participants described their eating patterns as “how I grew up eating,” and referred to meat as part of a “proper meal,” even among those motivated to reduce meat for sustainability reasons [30].

Security, defined as predictability and reassurance, also emerged as a decisive driver. During recent inflationary periods, consumers gravitated toward staple foods not only for affordability but also for their symbolic stability [22]. Reluctance to try novel proteins—owing to uncertainty about preparation, taste, and nutrition—was frequently reported [31], and even ethically motivated consumers expressed a need for reassurance when confronting unfamiliar protein sources. Overall, reluctance toward protein alternatives appears less a rejection of sustainability or health per se, and more a reflection of cultural continuity and affective safety.

Complementary to the published evidence, a series of exploratory focus groups conducted by the Fontys Research Group (Floto-Stammen) between 2023 and 2025 with students, educators, and company representatives (N ≈ 200) repeatedly identified Tradition and Security as dominant personal values guiding food-related attitudes and choices. While these sessions form part of an ongoing, unpublished practice-based project, they provide convergent indications that align with the reviewed literature and support the treatment of these two values as “given” anchors in the subsequent communication design. We treat this material as supporting grey evidence rather than as part of the formal review dataset.

We emphasise that these themes are not arbitrary labels but correspond to value dimensions explicitly used in the original studies’ operationalisations of Schwartz’s framework (e.g., items relating to family meals, home cooking, and inherited practices loading on Tradition; items relating to safety, reliability, and reassurance loading on Security). Given the small number and methodological heterogeneity of the included studies, a formal cross-study weighting of sub-items (e.g., by frequency or effect size) was not feasible. Instead, our synthesis focuses on convergent patterns across sources. We note that Sustainability and Health also emerged as relevant concerns but with less consistency across studies; in line with the reviewed evidence, we therefore treat Tradition and Security as primary value anchors and Sustainability and Health as important, yet secondary, motifs in the present analysis.

### 3.2. Communication Requirements

Findings from the synthesis were used to derive five communication requirements, identified through interpretive mapping of the two dominant values, Tradition and Security, onto food-related decision contexts. These requirements specify the emotional and behavioural conditions under which consumers may be more open to unfamiliar foods. Each requirement was grounded in thematic patterns observed across the reviewed studies that linked Tradition and Security to specific food-related attitudes and behaviours (see Table 2 for key supporting literature). In particular, the work of Koch et al., Hajdari, Hempel & Roosen, and Seffen & Dohle [22,23,30,31] draws on German data, whereas several studies cited for Emotional safety, Simplicity and clarity, and Trust and credibility in Table 2 provide conceptual but non-German evidence. To enhance transparency in the mapping, Tradition-related items (e.g., inherited food practices, family meal scripts, culturally “proper meals”) informed Cultural familiarity and Routine integration, whereas Security-related items (e.g., reassurance, risk avoidance, predictable outcomes) informed Emotional safety, Simplicity and clarity, and Trust and credibility.

If consumers’ food choices are anchored in cultural continuity and reassurance, communication needs to resonate with these underlying orientations. Cultural familiarity addresses Tradition by aligning new foods with recognisable formats, preparation practices, and sensory cues. Emotional safety reflects Security, focusing on reducing perceived risk and providing reassurance. Simplicity and clarity further support Security, ensuring that products appear easy to understand and integrate. Trust and credibility operationalise both values by relying on stable, reliable signals such as certifications and transparent labelling. Finally, routine integration corresponds to Tradition, emphasising everyday compatibility and continuity with established eating habits.

Recent packaging-communication research supports these requirements, showing that familiar and informative visual cues on packaging can reduce resistance such as disgust and enhance consumer trust toward insect-based and other alternative protein foods [26]. Earlier work on consumer acceptance among German and Dutch students likewise indicates that familiarity and clear information, reducing uncertainty and corresponding to the values of Tradition and Security, are key predictors of openness toward insect-based foods [32].

Together, these requirements define the emotional and behavioural conditions under which consumers may be more open to unfamiliar foods, providing the conceptual basis for designing values-centric communication.

### 3.3. Guidelines for Values-Centric Messaging

**Figure 2:**
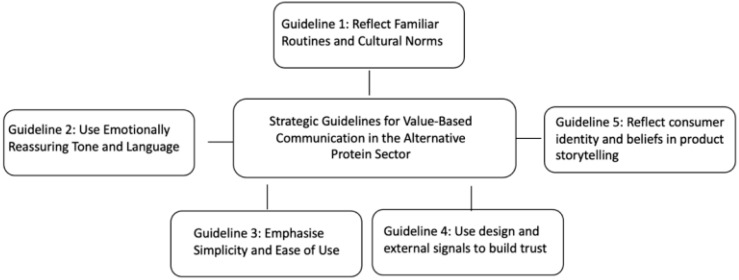
Values-centric communication guidelines for framing novel protein on packaging.

The five requirements were translated into values-centric communication guidelines, each grounded in the reviewed evidence and consistent with Schwartz’s value theory (See Figure 2). First, reflect familiar routines and cultural norms by presenting products in formats recognisable within German food culture (e.g., sausages, burgers, meatballs); acceptance increased when novel proteins resembled traditional formats [14,16]. Second, use an emotionally reassuring tone and language; wording associated with safety and predictability (e.g., “trusted,” “familiar”) tended to be better received, whereas technical or experimental phrasing elicited scepticism [20,22]. Third, emphasise simplicity and ease of use; consumers were more willing to adopt unfamiliar proteins when framed as “easy meal solutions” or accompanied by clear preparation cues [33,34]. Fourth, employ design and external signals to build trust; certifications, origin and traceability labels, and transparent claims supported perceived credibility, particularly among Security-oriented consumers, while overly futuristic aesthetics could signal risk [26,35]. Fifth, connect products to consumer identity; storytelling that situates products within family, heritage, or lifestyle elicited stronger resonance than purely technical specifications [36,37]. Collectively, these guidelines operationalise Tradition and Security into actionable principles.

**Table 2:** Communication requirements derived through interpretive mapping of Tradition and Security values across the reviewed literature.

Value	Factor		Requirements for the Guideline	Key Supporting Literature
Tradition	Cultural Familiarity		Connect with familiar food practices, traditions, and cultural expectations to create continuity.	Koch et al. (2021) [23], Hajdari (2023) [30], Naranjo-Guevara et al. (2020) [32]
Security	Emotional Safety		Create comfort, reassurance, and emotional ease, especially around novel or unfamiliar products.	Siddiqui et al. (2022) [20], Hempel & Roosen (2024) [22], Naranjo-Guevara (2020) [32]
Security	Simplicity Clarity	&	Use cognitively simple, low-effort messaging to reduce conflict or uncertainty.	Malek et al. (2023) [34]
Security	Trust Credibility	&	Signal reliability, transparency, and quality to reduce consumer doubt or uncertainty.	Wu et al. (2021) [35], Naranjo-Guevara et al. (2023) [26]
Tradition/Security	Routine Integration		Position the product as part of normal, habitual food behaviour to minimise disruption.	Michel et al. (2021) [14], Naranjo-Guevara et al. (2020, 2023) [26,32]

### 3.4. Application to Packaging Claims

The guidelines were applied to front-of-pack communication to demonstrate operationalisation at the point of purchase (Figure 2). Using a minimalistic reference pack [38] as a neutral baseline, five short claims were drafted according to operational rules derived from repeated patterns in the literature (Table 3). For instance, Cultural familiarity informed the claim “Your everyday crispy snack,” whereas Emotional safety informed “Trusted taste, made familiar.” This procedure ensured alignment of each claim with the values of Tradition and Security while maintaining a comparable visual balance across stimuli.

### 3.5. Consumer Responses

A single exploratory focus group (N = 7 German students) compared reactions to the original and refined packaging. Stimuli were presented in a fixed order (original then refined); order/contrast effects are acknowledged. To mirror McGuire’s framework, we summarise the findings by communication stage, from first glance (Attention) to self-reported intention (Action). Multiple elements were adjusted simultaneously in the refined version (imagery, headline, values-centric claims, trust icons), so responses reflect the combined stimulus. Analysis followed [25], which conceptualises persuasion as a sequence of five stages—attention, comprehension, acceptance, retention, and action. Participant comments were thematically organised according to these stages to trace how packaging cues shaped initial perception, understanding, trust, recall, and self-reported behavioural intention.

**Attention**. With the original pack, first fixation typically fell on the insect image or the term “insects” and was frequently accompanied by aversive affect (e.g., “disgusting,” “confusing”). A minority first noticed “protein snack” but then shifted attention to the insects. With the refined pack, initial attention moved to the product image/headline (“Crispy Protein Bites”); two participants spontaneously described the bites as “tasty.” Several did not recognise the insect base until reading further (e.g., “I had to look again to realize it’s insect-based,” Speaker 4). Some uncertainty about taste/texture remained. Overall, greater Cultural familiarity reduced negative first reactions and enhanced Emotional safety at a glance.

**Comprehension**. For the original, participants understood that the product was insect-based and high-protein, but the broader purpose or positioning was unclear and sometimes read as ironic/ambiguous (e.g., queries about whether it was intended as “comedy”). For the refined version, participants primarily interpreted it as a high-protein snack (fitness/health frame). Sustainability was inferred from claims (e.g., “eat for change,” “familiar taste”). A minority perceived ambiguity (e.g., bites seen as “raw”). In sum, Simplicity and clarity improved; partial masking of insect content increased neutrality but introduced some uncertainty.

**Acceptance** (credibility/trust). For the original, numeric claims (e.g., “52% protein”) and “Made in Germany” conveyed transparency/credibility, while affective unease about insects persisted (“trustworthy, but emotionally risky”). For the refined version, five participants reported higher trust due to a cleaner design, a certification icon, and origin labelling. Two, however, perceived information overload (“so much information… trying a little too hard”) and a claim–image mismatch (“doesn’t look crispy”), which reduced trust. Thus, credibility/trust improved via certification/origin, whereas Simplicity and clarity was undermined for some by dense text and visual–verbal incongruence.

**Retention.** For the original pack, the most frequently recalled elements were the insect image, the phrase “roasted cricket,” and the protein content; memorability of the image/headline skewed negative. A minority noted flavour descriptors (e.g., “lemon & pepper”), occasionally eliciting curiosity. For the refined pack, the certification icon “Certified”, “Crispy like home-cooked,” and “Eat for change without changing who you are” were commonly retained; the latter prompted self-reflection for one participant. The brand name “Catch Your Bug” continued to evoke aversive associations (e.g., “crawling insects”), counteracting otherwise reassuring cues. Identity-oriented phrasing supported Routine integration and Emotional safety when sufficiently salient; brand naming that primes insects undermined Emotional safety.

**Action** (self-reported intention). With the original pack, most participants stated they would not purchase; a few might try once in low-commitment contexts (e.g., social setting, discount) or if insects were non-visible (e.g., powdered). With the refined pack, more participants indicated they might consider purchase, citing familiar appearance, convenience framing (“on-the-go”), and reassuring tone. Some reported they would decline upon recognising the insect content; several noted that high information density reduced confidence. These shifts are consistent with improvements in Cultural familiarity and Emotional safety; persistent barriers centred on Simplicity and clarity surrounding insect disclosure.

**Claim-level reactions**. The headline “Crispy Protein Bites” (Guideline 1; Cultural familiarity) increased perceived familiarity (described as “snackable,” akin to common bite-sized snacks), though uncertainty about texture and flavour remained, indicating the need for stronger food-cue support. “Crispy like home-cooked. Familiar taste. Modern protein.” (Guideline 2; Emotional safety) was comforting for some; others questioned the specificity of “home-cooked” and noted incongruence between wording and imagery, which attenuated reassurance. “Great snack. On the go. Anytime.” (Guideline 3; Simplicity and clarity) communicated ease of use effectively; however, overall front-of-pack text density limited perceived simplicity. Trust signals “Made in Germany,” a certification icon, and “Traceable ingredients” (Guideline 4; Trust and credibility) differed in salience: certification/origin cues were salient and generally increased credibility, whereas “traceable ingredients” was rarely mentioned; several participants indicated that dense layouts reduced visibility of these cues and that the product’s insect content was not always clear. Finally, “Eat for change without changing who you are.” (Guideline 5; Routine integration/identity) was memorable and resonant when noticed; reduced typographic prominence limited exposure.

**Constraints specific to this test**. The focus group was small and student-based (N = 7); stimulus order was fixed (original then refined), introducing potential contrast/priming; and multiple elements were adjusted concurrently in the refined pack (imagery, claims, icons), limiting attribution to any single component. Purchase intentions were self-reported and hypothetical. Within these constraints, the observed movement from immediate aversion toward consideration is consistent with the central roles of Tradition (format familiarity, routines, identity) and Security (reassurance, credible signals, cognitive ease) identified in the synthesis.

## 4. Discussion

This study developed and preliminarily examined a values-centric communication approach for alternative proteins in Germany. Insect-based snacks served as our empirical test case because insect protein is discussed as a lower-impact alternative to conventional meat and thus as a potential contributor to more sustainable food systems, yet it faces particularly strong cultural and affective resistance in Western markets. Understanding how values-centric communication can reduce aversion and move consumers from rejection toward consideration for such products is therefore directly relevant to efforts to shift protein consumption in a more sustainable direction. A synthesis of four recent studies, combined with the authors’ own exploratory work with focus groups (N ≈ 200), identified *Tradition* and *Security* as the dominant value drivers of everyday food choice (see Section 3.1). From these, five communication requirements were derived: Cultural familiarity, Emotional safety, Simplicity and clarity, Trust and credibility, and Routine integration (Section 3.2). They were operationalised into guidelines (Section 3.3) and packaging claims (Section 3.4). Exploratory evidence from a single focus group (Section 3.5) indicated movement from immediate rejection toward consideration when messages and visuals increased familiarity and reassurance, while also revealing execution sensitivities (information load, claim–image mismatch, brand-name effects, and delayed recognition of insect content).

### 4.1. Theoretical Implications

First, the pattern of responses is consistent with the primacy of Tradition and Security in German food practices and with affect-first accounts of novel food avoidance. Early reactions were strongly emotional (e.g., disgust or aversive affect), aligning with classic work on disgust and food neophobia [17] and with the affect heuristic in risk perception, which suggests that people rely on affective impressions rather than analytical reasoning when evaluating novel risks [39]. Strengthening Cultural familiarity (recognisable formats, non-threatening visuals) and Emotional safety (reassuring tone) coincided with improvements at early persuasion stages (Attention, Comprehension), which is consistent with low-elaboration processing models in which peripheral cues guide attitude formation [25].

Second, the data underscore the importance of cue congruence and processing fluency. Perceived inconsistency between the “crispy” claim and visual texture, as well as high front-of-pack (FOP) text density, coincided with weaker Simplicity and clarity and Trust and credibility. This pattern is consistent with evidence from advertising research showing that congruent visual–verbal cues enhance memory and persuasiveness [40] and with the concept of processing fluency, whereby low complexity and clear visual hierarchy facilitate liking and perceived truth [41].

Third, Routine integration maps onto habit theory: food choices are often guided by stable scripts and contexts rather than deliberate decision-making, as shown in habit-based models of consumer behaviour [42]. Positioning novel proteins to fit existing routines therefore provides a mechanism by which Tradition and Security values can be activated and maintained over time. Finally, the observed salience of certifications and origin labels aligns with work on credence attributes and consumer trust in food [26,35], while identity-linked phrasing reflects findings from research on narrative processing and self–brand connections, which demonstrate that messages consistent with the consumer’s self-concept enhance engagement and acceptance [43,44].

Together, these findings support the integration of Schwartz’s values framework with stage-based persuasion theory, suggesting that value activation (Tradition/Security) can be mapped onto stage-specific communication tasks (e.g., clarity for Comprehension; credible signals for Acceptance; identity resonance and routine fit for Retention). More specifically, Schwartz’s framework provides the motivational content of communication (what higher-order goals messages should speak to), whereas McGuire’s sequence specifies the process (when in the persuasion trajectory each goal is most critical and which psychological tasks must be solved at that point).

In our data, Tradition primarily manifests in the requirements of Cultural familiarity and Routine integration: familiar product formats and culturally recognisable cues help to secure early-stage Attention and Comprehension, while embedding novel proteins into existing eating scripts supports Retention and repeated use. Security is most clearly expressed through Emotional safety and Trust and credibility: non-threatening framings and reassuring language help to regulate initial affect at the Attention stage, whereas concrete, verifiable cues (e.g., certifications, origin labels) foster Acceptance (yielding) and longer-term trust. Simplicity and clarity function as a cross-cutting requirement that serves both Tradition (preserving familiar interpretive frames) and Security (reducing perceived complexity and risk) and is particularly central at the Comprehension and Acceptance stages. This crosswalk illustrates how values (Tradition, Security) can be translated into stage-specific communication tasks (Attention, Comprehension, Acceptance, Retention), thereby operationalising the combination of Schwartz and McGuire into a coherent values-centric communication framework for alternative proteins. At the same time, comparison with prior work on alternative proteins, packaging cues, and values-based communication indicates that our framework should be regarded as an initial, incomplete model that requires further empirical testing and refinement. This stage-based mapping complements dual-process perspectives, which distinguish between affective/peripheral and analytic/central processing, by specifying the sequence of tasks (attention, comprehension, acceptance, retention) through which value cues must pass in packaging contexts. It also extends existing value-framing work by not only identifying which values (e.g., Tradition, Security) are relevant, but also clarifying at which persuasion stages they are likely to be most influential (e.g., reassurance at Attention/Comprehension; identity and routine fit at Acceptance/Retention). In that sense, our framework links value content and persuasion process more tightly than prior studies that focus mainly on value congruence at the message level.

### 4.2. Managerial Implications

Drawing on the synthesis and the exploratory focus-group evidence, several implications for practice emerge. First, strengthening cultural familiarity by presenting novel proteins in recognisable formats and ensuring claim–image congruence (e.g., visual texture consistent with “crispy”) appears to reduce initial aversion and support early-stage processing, consistent with established fluency and congruence effects [40,41]. Second, emotional safety is fostered by a reassuring tone and by avoiding technical or experimental framings that may elevate perceived risk under low elaboration [45,46]; conversely, brand names that foreground insects can prime disgust and counteract reassurance [15]. Third, simplicity and clarity are enhanced when FOP information is limited to a primary message with a clear visual hierarchy, reserving detail for secondary panels; excessive textual density undermines fluency and perceived credibility [47]. Beyond perceptual and cognitive fluency, trust and identity-based mechanisms further support acceptance. Fourth, trust and credibility benefit from salient, concrete cues (e.g., certification marks, origin labels), consistent with findings from [26], whereas abstract phrasing (e.g., “traceable ingredients”) showed low salience without specific verifiable anchors. Finally, routine integration and identity alignment are supported when identity-linked claims are typographically prominent and narratively coherent, aiding recall and normalising adoption without threatening self-concept, which is in line with the concept of self-congruity and identity-based persuasion [43,44]. These implications remain exploratory and should be treated as hypotheses for validation in larger, controlled studies. At the same time, any move toward more familiar imagery or partial masking of insect content must remain fully compatible with regulatory requirements for ingredient and allergen disclosure. Insect proteins can be related to shellfish allergies, and packages must clearly communicate insect content and relevant allergen information. Practitioners should therefore treat reassurance-oriented design as a complement to, rather than a substitute for, transparent labelling and legal compliance.

### 4.3. Limitations

This study has several limitations. First, interpretation is constrained by the very small and homogeneous student sample (N = 7 from a single university). The sample was limited to English-fluent German students, which restricts representativeness and introduces potential selection bias. Therefore, findings should not be generalised beyond similar demographic groups. Given this single focus group, theoretical saturation was neither sought nor claimed; the qualitative findings should be interpreted as hypothesis-generating. Future studies should therefore use larger and more diverse samples that include consumers of different ages, educational backgrounds, and regions. Second, the refined packaging stimulus was created with generative AI and implemented several changes simultaneously (imagery, multiple front-of-pack claims, icons). As a result, any observed differences between the original and refined versions cannot be attributed to a specific element, and stylistic artefacts typical of AI-generated artwork may have influenced participants’ perceptions. Future research should use professionally designed stimuli and factorial experimental designs (e.g., systematically varying imagery, claims, and icons) to tease apart the unique and interactive effects of individual packaging components. Also the refined packaging was always shown after the original version, introducing a possible sequence/contrast effect. A counterbalanced or randomized presentation order is recommended for future studies. Third, purchase intentions were hypothetical rather than observed behaviour. Finally, the reference brand name itself introduced a persistent negative prime. These limitations are typical for exploratory qualitative work and highlight the need for the confirmatory studies described below. In addition, restricting the literature synthesis to 2021–2025 may have excluded earlier foundational work on values and food choice, limiting the breadth of the evidence base. The internal Fontys focus groups used for triangulation are unpublished, practice-based sessions and are treated as grey evidence rather than formal data.

### 4.4. Future Research

Future work should employ randomised, counterbalanced designs; factorial experiments to isolate the effects of imagery, tone, trust iconography, brand naming, and disclosure; larger and more diverse samples; and behavioural endpoints (e.g., choice tasks, field A/B). Research should also test values-based segmentation (weighting guidelines by audience) and evaluate transparent yet non-aversive disclosure of insect content (placement/wording) in light of known disgust/neophobia dynamics.

## 5. Conclusions

This study proposed and preliminarily examined a values-centric communication approach for alternative proteins in Germany. A values-centric approach foregrounding Tradition and Security, operationalised through Cultural familiarity, Emotional safety, Simplicity and clarity, Trust and credibility, and Routine integration, appears to reduce initial aversion and move German consumers toward consideration of alternative proteins. Exploratory focus-group evidence suggests that familiar formats, a reassuring tone, a clear visual hierarchy, and salient trust cues may improve early-stage responses, whereas information overload, claim–image incongruence, and value-incongruent naming impede acceptance. The study contributes an integrative analytic lens combining Schwartz’s value theory with McGuire’s communication–persuasion model, and a set of testable guidelines for values-aligned food communication. However, the empirical evidence presented here is explicitly preliminary. Given the exploratory design, small and homogeneous student sample, hypothetical purchase intentions, and AI-generated stimuli that bundled multiple design changes, findings should be regarded as hypothesis-generating rather than definitive; robust validation using counterbalanced, factorial, and behavioural designs with larger and more diverse samples remains an important priority for future research.

## Figures and Tables

**Table 1:** Core personal values influencing eating behaviour in Germany (summary of reviewed studies).

Study	Values Highlighted	Key Findings	Consumer Segment
Koch et al. (2021) [23]	Tradition	Meat seen as essential part of a “proper” meal for 97% of consumers	General adult population
Hajdari (2023) [30]	Tradition	Food habits tied to upbringing and identity	Young adults (qualitative sample)
Hempel & Roosen (2024) [22]	Security	Trusted everyday foods preferred during inflation for their reliability and simplicity	Broad demographic sample
Seffen & Dohle (2023) [31]	Security	Hesitation linked to uncertainty about nutrition, preparation, and taste	Health-conscious individuals
Fontys (Floto-Stammen, 2023–2025) ongoing values-study (unpublished)	Tradition & Security	Emotional reliance on routines even among sustainability-minded consumers.	Flexitarians, omnivores

**Table 3:** Operational rules for claim development and examples of values-centric claims.

Guideline	Operational Rules Applied	Claim
G1: Reflect familiar routines and cultural norms	Rule 1: Use culturally familiar language that aligns with everyday food categories and terminology.	*Crispy Protein Bites* *(renamed from Insect* *Protein Snack)*
G2: Emotionally reassuring tone and language	Rule 2: Use emotionally reassuring adjectives and avoid experimental or disruptive language.	*Crispy like home-cooked. Familiar taste. Modern protein*
G3: Emphasize simplicity and ease of use	Rule 3: Keep phrasing short and show the snack is quick and easy to consume.	*Great snack. On the go. Anytime.*
G4: Use design and signals to build trust	Rule 4: Apply trust-building cues and icons through both wording and icons.	*Made in Germany, certification icons, Traceable ingredients*
G5: Reflect consumer identity in product storytelling	Rule 5: Frame claims to reflect consumer identity	*Eat for change without changing who you are*

## Data Availability

The original contributions presented in this study are included in the article. Further inquiries can be directed to the corresponding author. The anonymized transcript of the exploratory focus group conducted for this study is openly available in DataverseNL at https://dataverse.nl/dataset.xhtml?persistentId=doi:10.34894/ZQGWSL, accessed on 10 December 2025.
